# Solid-State NMR Spectroscopy: A Key Tool to Unravel the Supramolecular Structure of Drug Delivery Systems

**DOI:** 10.3390/molecules26144142

**Published:** 2021-07-07

**Authors:** Marianna Porcino, Xue Li, Ruxandra Gref, Charlotte Martineau-Corcos

**Affiliations:** 1 CEMHTI UPR CNRS 3079, Université d’Orléans, 45071 Orléans, France; 2 Institut des Sciences Moléculaires d’Orsay, UMR CNRS 8214, Paris-Sud University, Université Paris Saclay, 91400 Orsay, France; xue.li@universite-paris-saclay.fr (X.L.); ruxandra.gref@universite-paris-saclay.fr (R.G.); 3 CortecNet, 7 Avenue du Hoggar, 91940 Les Ulis, France

**Keywords:** solid-state NMR spectroscopy, porous material, drug delivery system, heteronuclei

## Abstract

In the past decades, nanosized drug delivery systems (DDS) have been extensively developed and studied as a promising way to improve the performance of a drug and reduce its undesirable side effects. DDSs are usually very complex supramolecular assemblies made of a core that contains the active substance(s) and ensures a controlled release, which is surrounded by a corona that stabilizes the particles and ensures the delivery to the targeted cells. To optimize the design of engineered DDSs, it is essential to gain a comprehensive understanding of these core–shell assemblies at the atomic level. In this review, we illustrate how solid-state nuclear magnetic resonance (ssNMR) spectroscopy has become an essential tool in DDS design.

## 1. Introduction

**Figure 1:**
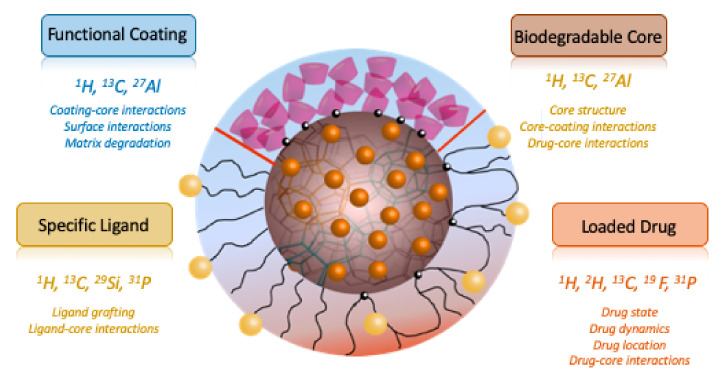
Scheme of a DDS nanoparticle containing a core, in which the drug is loaded, functional coating, and specific ligand. The nuclei of interest in ssNMR and the information that can be extracted from these measurements are indicated.

A nanosized drug delivery system (DDS) is defined as a formulation or a device that enables the introduction of a therapeutic substance in the body [1]. The major goals of DDS are to improve the efficacy and safety of a given active pharmaceutical ingredient (API) by controlling its rate, time, and place of release as well the compliance of the patient [2]. A plethora of nanosized DDSs were developed, including liposomes, polymeric and inorganic nanoparticles, dendrimers, carbon nanotubes, etc. However, there is no universal drug nanocarrier, and for each medical target, a new system has to be designed. An ideal DDS should have high colloidal stability, high drug loading capacity, be prepared without the need for toxic solvents, be inert, biocompatible, mechanically resistant, biodegradable, well tolerated by the patient, safe and simple to administer, and finally easy and cost-effective to fabricate and sterilize [3]. Most DDSs are composed of complex core–shell supramolecular structures where each component has different physicochemical characteristics and functions (Figure 1). The core of the system accommodates the drug, protects, and releases it in a controlled manner. It must ensure efficient drug release and degrade to avoid accumulation in the body. The corona plays a role in increasing the colloidal stability of the nanoparticles and governs their in vivo fate (confers “stealth” properties to evade the immune system and/or to ensure specific delivery to the biological target). To guide the design of complex core–shell particles, it is essential to have at hand analytical tools able to yield information at the atomic scale about the structure of the DDS, the host–drug interactions, dynamics, etc.

A variety of techniques have been used so far, such as thermal analysis [4] and nitrogen sorption [5], which can both give qualitative and quantitative information about the bulk structural and thermodynamic properties of the incorporated drugs. Unfortunately, they do not provide any insight into the microscopic properties of the embedded drugs and in particular about the crucial drug–drug and drug–host interactions. X-ray powder diffraction (XRPD), which is the usual method of choice for the investigation of structural properties, is also often inapplicable because of the lack of long-range order of complex DDSs. An alternative technique is solid-state nuclear magnetic resonance (ssNMR), which through spectroscopy and relaxation measurements offers valuable information about the structural and dynamical properties of the embedded compound and which is not limited to the materials or forms that exhibit long-range order [6]. ssNMR experiments probe the local environment of the nucleus and short-range order. This gives rise to various advantages, including the ability to provide detailed structural insights from polycrystalline samples and to examine local defects and disorders [7].

In the context of DDSs, because numerous NMR active nuclei are present in both the core and the corona, ssNMR spectroscopy can provide information in many aspects ranging from intramolecular and intermolecular interactions, localization, state and stability of the drug, core–shell interactions, the matrix degradation, delivery/release of the drug, etc. (Figure 1). These pieces of information can significantly contribute to guide the design and development of new DDSs. Moreover, ssNMR is non-destructive; i.e., the DDS can be studied intact without the need for dissolution/digestion (as it would be required for solution-state NMR, fluorescence spectroscopy or other strategies involving chemical modification).

In this review, we illustrate the versatility of ssNMR spectroscopy for the investigation of the supramolecular structure of DDSs. We focus this review on DDSs that are based on inorganic or hybrid organic–inorganic particles, i.e., rather than on organic particles such as liposomes or polymer-based ones. We first report ssNMR experiments based on one-dimensional (1D) NMR spectroscopy, then two-dimensional (2D) NMR starting from the simpler ^1^H-^1^H spin pair, then moving toward ^1^H-X pairs and finally to more challenging pairs of heteronuclei. For each case, the advantages and limits of the ssNMR technique are described.

## 2. A Few Notes on ssNMR Spectroscopy

An ssNMR spectrum, similarly to a solution-state NMR spectrum, contains information about the chemical shift interaction, i.e., a line shift that gives indication about the neighboring environment of a nucleus, and, when it can be observed or detected, about scalar coupling, i.e., a line splitting, which represents the spin–spin interaction through chemical bonds and gives information about the number and nature of neighboring nuclei [8,9]. In the solid state, two other interactions are expressed in the ssNMR spectra: the dipolar interaction, that is a through-space interaction between nuclei, and the quadrupolar interaction for atoms with nuclear spin quantum numbers larger than ½. The former is mostly responsible for an important homogeneous line broadening of ssNMR resonances as compared to solution NMR resonances (for which this interaction is averaged to zero due to fast molecular tumbling), the second can give rise to particularly broad NMR spectra. Magic-angle spinning (MAS), i.e., spinning the sample at an angle of 54.7° about the main—usually vertical—magnetic field is the most used method to remove the dipolar interaction, that is the major source of resolution loss for solids. The faster the spinning, the most efficient the removal of the interaction [10].

## 3. 1D ^1^H ssNMR Spectroscopy

In the world of DDSs, mesoporous silica nanoparticles (MSNs) are among the most studied systems. Their long-range ordered pore structure with tailorable pore size (2–50 nm) and geometry facilitates a homogenous incorporation of guest molecules with different sizes and properties. Their surface can further be easily functionalized. Drug loading is based on the adsorptive properties of MSNs and both hydrophilic and hydrophobic cargos can be adsorbed into the pores. The release profile of drugs from MSNs mainly depends on its diffusion from the pores, which can be tailored by modifying the surface of the MSNs to suit the biological needs. The decisive factor responsible for controlling the release is the interaction between the surface groups on pores and the drug molecules. The two most widely explored materials for drug delivery are MCM-41 (Mobil Crystalline Materials) and SBA-15 (Santa Barbara Amorphous type material) [11,12]. MSNs owing to their modifiable surface chemistry can act as carriers for poorly soluble drugs and tackle their solubility issues. ^1^H, ^13^C, and ^29^Si are the most used nuclei to study the interactions between silica molecules and drugs and the influence in the environment of silicon atoms in SBA-15 [13,14,15,16].

^1^H is indeed the most obvious nucleus that can be used for the characterization of DDS as the majority of drugs are protonated, as are the drug carriers. 1D ^1^H MAS NMR is a simple and fast experiment that gives information about the state of the drug in the DDS and the formation of hydrogen bonds (that leads to high-frequency proton shifts), thanks to a shift of the proton resonance. The spectra are known to be dominated by large homonuclear dipolar interactions; hence, techniques based on fast MAS are usually preferred to obtain high-resolution data [17]. Due to the small chemical shift dispersion of ^1^H NMR spectra (below 20 ppm in most diamagnetic systems), it is preferable to perform measurements at high magnetic field to optimize the signal resolution.

**Figure 2:**
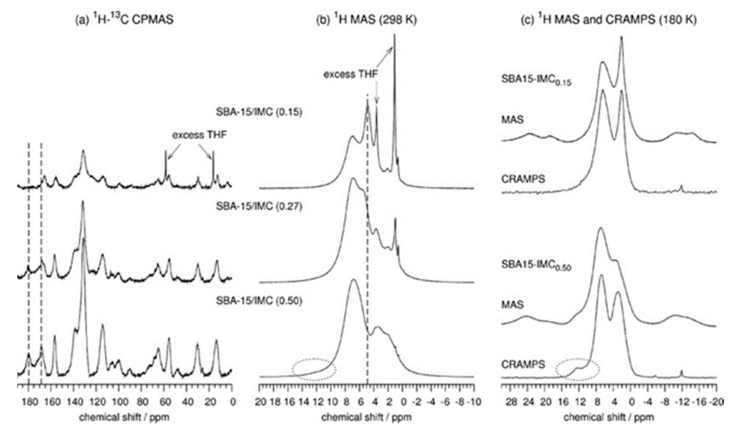
^1^H-^13^C CPMAS (**a**), ^1^H MAS (**b**), ^1^H MAS, and CRAMPS (**c**) experiments of SBA-15 in which were loaded different amount of indomethacin [6]. (Reprinted with permission from T. Ukmar, T. Čendak, M. Mazaj, V. Kaučič, G. Mali, J. Phys. Chem. C 2012, 116, 2662. Copyright © 2012, American Chemical Society).

Ukmar et al. [6] studied by ssNMR the composition and structure of nonfunctionalized and functionalized SBA-15 mesoporous silica matrices in which were loaded different amounts of indomethacin (IMC) molecules. From the ^1^H MAS NMR spectra (Figure 2), a decrease in the signal intensity of the water resonance was detected when the amount of the drug was increased. Furthermore, the appearance of a new resonance revealed the formation of hydrogen bonds between the drug and the silica matrix. These were evidences for drugs in close contact in the pores with the silica matrix.

**Figure 3:**
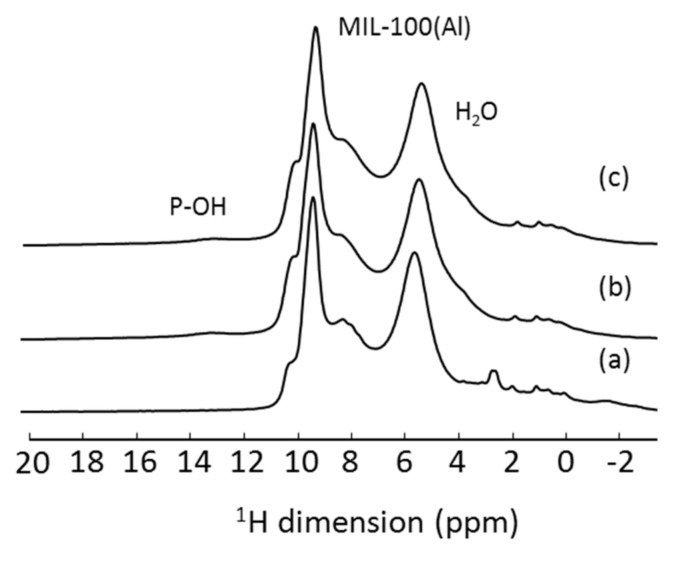
^1^H MAS NMR spectra of (**a**) nanoMIL-100(Al), (**b**) ATP-loaded nanoMIL-100(Al) and (**c**) CD-P coated ATP-loaded nanoMIL-100(Al).

However, in DDSs, most systems are complex and lacking long-range order; therefore the resonances are strongly heterogeneous, and even using the fastest available MAS probes and highest static magnets is not enough to obtain satisfactory resolution. This is exemplified in Figure 3 for a model DDS system consisting of an aluminum-based nanoscale metal–organic framework (MOF). This MOF, MIL-100(Al) (MIL stands for Matériau Institut Lavoisier), was analyzed pure, loaded with a drug and with surface covered by β-cyclodextrin (CD) phosphates. The differences between all three samples are not easily seen on the 1D MAS (60 kHz, 20.0 T) ^1^H NMR spectra, due to strong overlap between all the components, each of them containing a fairly large number of protons. Two solutions might be adopted to overcome this problem: performing a two-dimensional (2D) homonuclear experiment (illustrated in the next section) or the use of deuterated molecules.

## 4. ^1^H-^1^H 2D NMR

**Figure 4:**
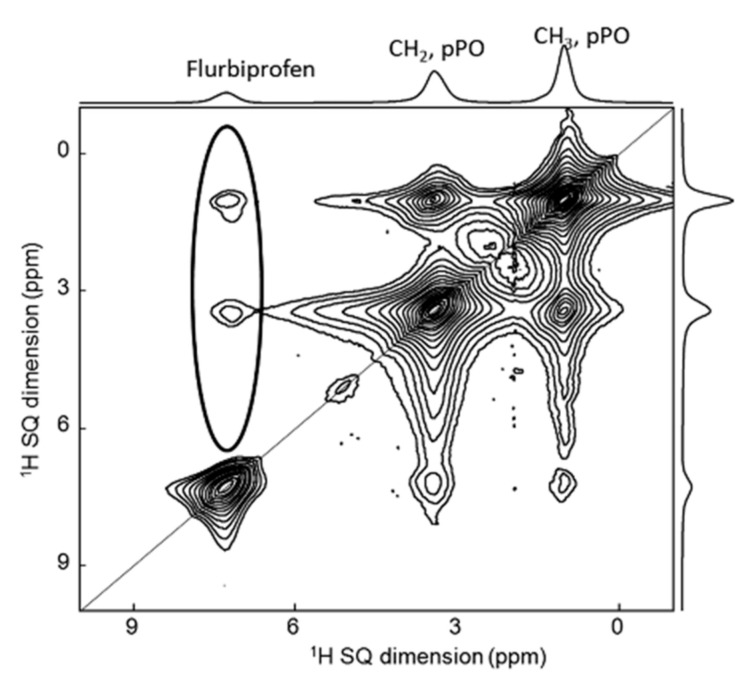
^1^H-^1^H 2D SQ-SQ MAS NMR of flurbiprofen incorporated in silica capsules filled with Pluronic P123. As evidence (ringed), the cross-peaks presented between the protons of the drug and the ones of the DDS [18].

The amount of information from 1D NMR spectra is sometimes limited due to potential overlap on the resonances. Therefore, 2D NMR spectroscopy is often used for characterization in pharmaceutical analysis [17]. As an example, it was employed to study flurbiprofen incorporated in 200–400 nm silica capsules filled with Pluronic P123 (polyethylene oxide-polypropylene oxide-polyethylene oxide triblock copolymer). ^1^H-^1^H 2D single-quantum single-quantum (SQ-SQ) NMR experiments (Figure 4) revealed the close proximity between the protons of flurbiprofen molecules and those of polypropyleneoxide part of the P123 chains. This confirmed the solubilization of flurbiprofen inside the core of the micelles composed of poly(propylene oxide), and its absence from the shells made of poly(ethylene oxide) [18]. 

**Figure 5:**
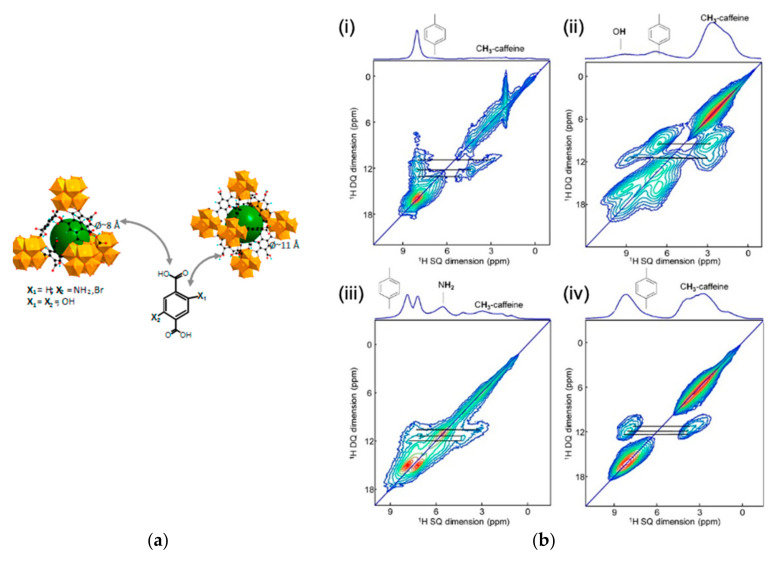
(**a**) Schematic view of the tetrahedral (left) and octahedral (center) cages of the dehydrated UiO-66(Zr). Zirconium polyhedra and carbon atoms are in orange and black, respectively. Hydrogen atoms are omitted for clarity. (**b**) ^1^H-^1^H DQ-SQ MAS NMR experiments on different functionalized UiO-66(Zr) samples (-H (**i**), -NH_2_ (**ii**), -2OH (**iii**), -Br (**iv**)) loaded with caffeine. The lines indicate the correlation between the protons of the linker of the MOF and the ones of the CH_3_ group of the caffeine [21].

Another important class of porous nanosized DDS are MOFs [19], which are based on metal clusters linked to each other through organic linkers. They offer highly porous structures that can accommodate, transport, and release drugs. The most promising MOFs are based on non-toxic paramagnetic Fe^3+^ cations, which limits the NMR investigations [20]. Indeed, ssNMR offers more information when diamagnetic cations such as Ca^2+^, Zn^2+^, Al^3+^, or Zr^4+^ are used. As an example, UiO-66(Zr), where UiO stands for the University of Oslo, has been tested as a carrier for caffeine [18]. The terephthalate linker can be functionalized with different polar/apolar groups, which can allow tuning of the host/guest interactions. ^1^H and ^13^C ssNMR experiments were performed to investigate these interactions. The interactions between the MOFs and the drug were studied by combining density functional theory (DFT) calculations and ^1^H-^1^H double-quantum single-quantum (DQ-SQ) MAS NMR experiments. To do so, different functionalized UiO-66(Zr) samples (-H, -NH_2_, -2OH, -Br) loaded with caffeine were used (Figure 5). Notably, they show that the functional groups had little impact on the drug as no specific interaction between the caffeine and the functional group was found on the NMR spectra [21].

**Figure 6:**
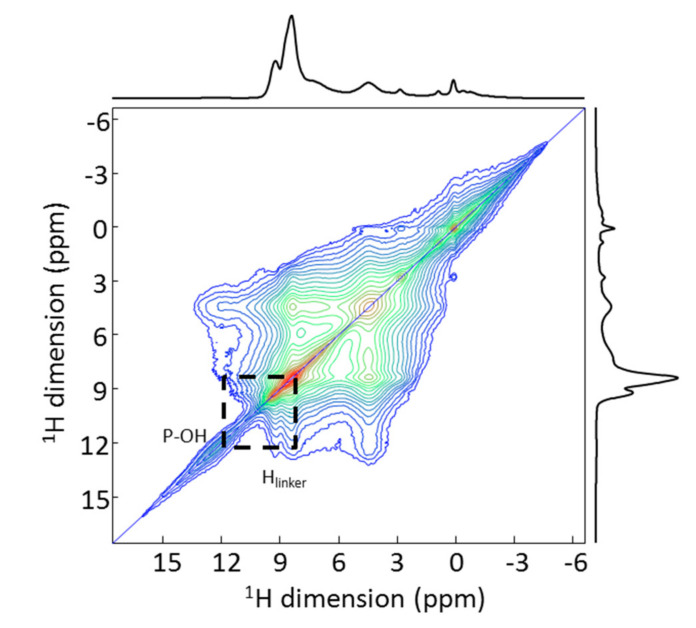
2D ^1^H-^1^H spin diffusion NMR spectrum of CD-P coated ATP-loaded nanoMIL-100(Al). The dash lines indicate the spatial proximity between a phosphate proton and a proton from the linker of the MOF.

Coming back to the example shown in the previous section (MIL-100(Al) coated with cyclodextrin-phosphate and loaded with adenosine triphosphate (ATP), for which no resolution was obtained on the 1D MAS NMR spectrum, one can notice a slightly better resolution of the resonances on the 2D ^1^H-^1^H MAS NMR (Figure 6). In particular, a correlation peak between the phosphate group of the drug and the proton of the MOF linker is observed, confirming the incorporation of the drug in the pores of the MOF. However, the overlap is still very strong, and it is difficult from this spectrum to extract further unambiguous correlation patterns.

## 5. ^1^H-X NMR

An alternative solution to address the challenges of analyzing the structure DDSs is to take advantage of the heteroatoms (i.e., non-hydrogen atoms) when they are present in the drug or in the host. Hereafter, we illustrate applications of ^1^H-X ssNMR spectroscopy in this context.

^13^C nucleus is the second most obvious nucleus to study by NMR, as most drugs but also numerous DDSs contain carbon atoms, either in the grafted molecules or in the host structure itself (e.g., organic DDSs, MOFs, or functionalized porous silica). Solid dispersions can be analyzed by ^13^C ssNMR to probe the association between amorphous drug and polymers through differences in NMR spectra that are not visible in the PXRD pattern [22,23] or to provide a direct way to probe drug–carrier interactions [24,25,26] and analyze the polymorphic forms of drugs [27], or to distinguish between the free and bound steroid drug in a DDS [28]. For example, in solid dispersions formed by α-, β-, and γ-CD in polyethylene glycol (PEG) 6000 g/mol with or without the addition of 5% *w*/*w* indomethacin (IM), the ^13^C cross-polarization under MAS (CPMAS) NMR spectra of the α- and β-CD solid dispersions gave spectra that were essentially superpositions of the spectra of the pure components of the system. On the contrary, for the γ-CD based dispersion, the spectral resolution was somewhat better, and therefore, several chemical shift data for C-1, C-4, and C-6 of the CDs were obtained. They concluded from these data that an inclusion complex might have been formed where the PEG molecules or their hydroxyl end groups interact with the CD cavity, chasing the bound water molecules from the cavities and changing the chemical shift in a way similar to that obtained when water was present. In the PEG/IM, PEG/α-CD/IM, and PEG/β-CD/IM samples as in pure IM spectra, these two peaks are of the same magnitude, while the C-10 peak has almost disappeared in the γ-CD dispersion (PEG/γ-CD/IM). For γ-CD, C-1 and C-4 signals showed a shift of 1–4 ppm downfield, while C-2,3,5 signals showed a shift of 1 ppm in the opposite direction compared to pure γ-CD. The C-6 carbon located at the exterior of the torus also showed a shift of 2 ppm upfield. These results indicate that IM may interact with γ-CD not only at the interior of the cavity but could also affect, directly or indirectly, the hydrogen bonds at the top of the torus where C-6 is located [29].

**Figure 7:**
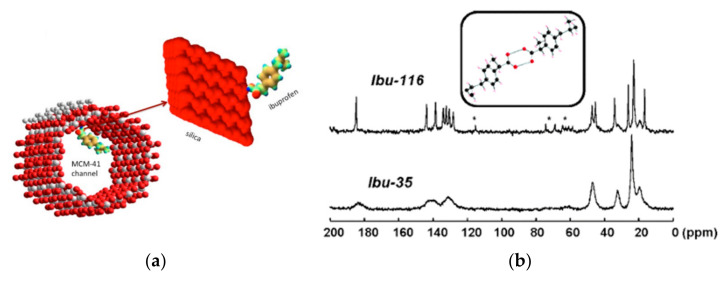
*(***a**) Adsorption of a molecule of ibuprofen within the MCM-41 channels by electrostatic interactions; (**b**) ^13^C CPMAS NMR spectra of Ibu-116 and Ibu-35 [30]. The stars indicate the presence of spinning sidebands. (Reprinted with permission from T. Azaïs, C. Tourné-Péteilh, F. Aussenac, N. Baccile, C. Coelho, J.-M. Devoisselle, F. Babonneau, Chem. Mater. **2006**, 18, 6382. Copyright © 2006, American Chemical Society).

The surface of MCM-41 was modified with silane or other organic or amino groups [14,15,16,17] with the aim to better control the drug release. Azais et al. [30] reported a study on ibuprofen (Ibu), loaded in MCM-41 with pore size ranging from 35 to 116 Å (Figure 7a). Using ^1^H, ^13^C, and ^29^Si ssNMR experiments recorded at room or low temperature (−50 °C), the authors clearly showed the local interactions between the drug and the MCM-41 host, which they could further relate to the drug release profile. Figure 7b displays the ^13^C CPMAS NMR spectra of Ibu-116 (Ibu loaded in MCM-41 with pore size of 116 Å) and Ibu-35 (Ibu loaded in MCM-41 with pore size of 35 Å) recorded at −50 °C. The two spectra are very distinct; in particular, if the spectrum of Ibu-116 is close to the one of the crystalline Ibu, the one of Ibu-35 presents broader peaks. The pores with diameters of 116 Å are large enough to allow the nucleation of Ibu crystallites. In contrast, in narrow pores (35 Å), a vitrification process occurs as demonstrated in the ^13^C NMR spectrum (Figure 7b), in which the quaternary carbons are now clearly detected [30].

**Figure 8:**
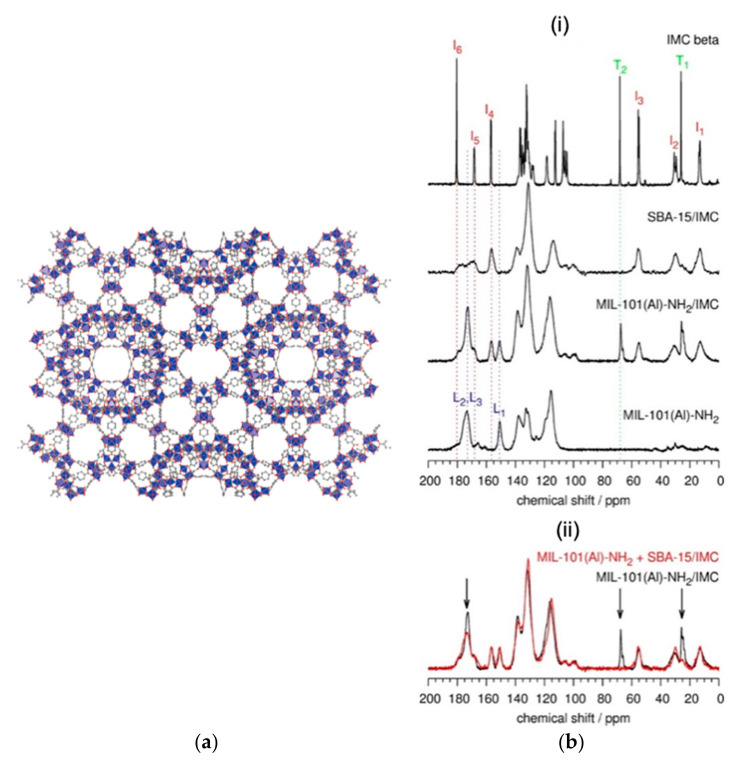
(**a**) Structure of MIL-101 viewed along the (101) direction. (**b**) (**i**) ^1^H−^13^C CPMAS NMR spectra of bulk crystalline β- IMC, SBA-15/IMC, loaded and empty MIL-101(Al)-NH_2_. Peak labels correspond to selected carbon atoms within the IMC molecule (I1−I6), THF molecule (T1, T2), and BDC-NH_2_ linker (L1−L3). Vertical dotted lines enable an easier comparison of signal positions. (**ii**) Comparison of the ^1^H−^13^C CPMAS NMR spectrum of MIL-101(Al)-NH_2_/IMC with the sum of the spectra of SBA-15/IMC and MIL-101(Al)-NH_2_. Arrows indicate details where the differences are the most pronounced [31]. (Reprinted with permission from T. Čendak, E. Žunkovič, T. Ukmar Godec, M. Mazaj, N. Zabukovec Logar, G. Mali, J. Phys. Chem. C **2014**, 118, 6140. Copyright © 2014, American Chemical Society).

β-IM was loaded in the cavities of both MIL-101(Al)-NH_2_ and a mesoporous silica SBA-15 (Figure 8a). Detailed inspection of the ^1^H-^13^C CPMAS NMR spectrum of MIL-101(Al)-NH_2_/IM (Figure 8b) leads to two interesting observations. First, unlike the spectrum of SBA-15/IM, the spectrum of MIL-101(Al)-NH_2_/IM clearly confirms the presence of tetrahydrofuran (THF) within the pores. Second, the carboxylic carbon peak at 173 ppm (named L2, L3 in the spectrum) narrows substantially. This suggests that the metal–organic framework undergoes structural ordering when it is filled with IM molecules [31].

Another interesting nucleus is ^19^F, as about 25% of APIs present on the market contain a fluorine atom in their structure [32]. With a sensitivity close to that of proton, ^19^F is highly attractive from an NMR point of view. It is usually present in little quantity in the API (often less than two or three fluorine groups), leading to ^19^F MAS NMR spectra with a limited number of resonances. Furthermore, the chemical shift dispersion (but also the chemical shift anisotropy) is much larger than that of proton, leading to less signal overlap. It can provide information also on the molecular orientation inside a DDS [33].

**Figure 9:**
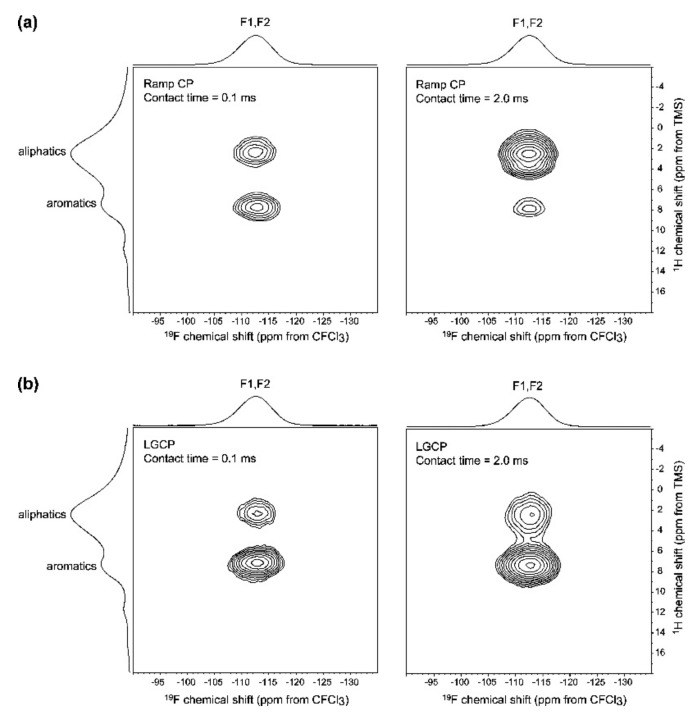
(**a**) ^1^H-^19^F CP-HETCOR spectra of a 30% w/w amorphous dispersion of diflunisal (VI) in PVP; (**b**) ^1^H-^19^F LGCP-HETCOR spectra obtained under the same conditions, except with the use of LGCP to suppress spin diffusion. The difference in relative correlation intensity with a 2.0 ms contact time between the two CP methods highlights the magnitude of the spin diffusion effects between VI and PVP in the dispersion. The F2 projections are the ^19^F CP-MAS spectrum recorded at 15 kHz), and the F1 projections are the ^1^H MAS spectrum recorded at 35 kHz) [22]. (Reprinted with permission from T. N. Pham, S. A. Watson, A. J. Edwards, M. Chavda, J. S. Clawson, M. Strohmeier, F. G. Vogt, Mol. Pharm. **2010**, 7, 1667. Copyright © 2010, American Chemical Society).

Pham et al. [22] performed ^1^H-^19^F CP-HETCOR and Lee-Goldburg (LG) CP-HETCOR experiments (Figure 9) on acetaminophen amorphous dispersions in poly(vinyl pyrrolidone) (PVP) to confirm the formation of an amorphous glass solution. Note that to perform this type of experiment, a particular HFX triple resonance probe design is required [34]. Correlations are observed between the fluorine signal and both the types of protons (aromatic and aliphatic). The aliphatic ones can only be associated with the polymer. Increasing the contact time (2 ms), the correlation increases as expected from spin diffusion. To confirm spin diffusion effects, the authors performed LGCP-HETCOR experiments, which greatly reduces spin diffusion during the ^1^H spin lock period (Figure 9b). As shown by the relative intensity of the correlations, the build-up of spin diffusion is eliminated; the remaining correlation between the fluorine signals and aliphatic protons can then be assigned to a direct through-space dipolar interaction.

**Figure 10:**
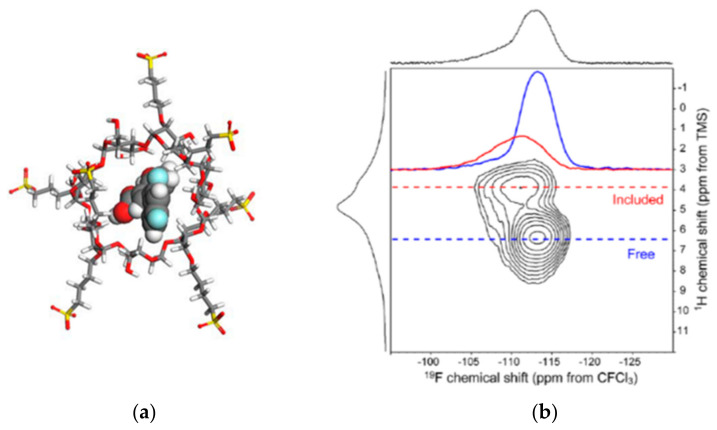
(**a**) Structure of diflunisal and β-CD complex; (**b**) ^1^H−^19^F CPHETCOR spectrum showing a mixture of bound and unbound diflunisal. In the F2 axis dimension is plotted the ^19^F CP-MAS spectrum (at 14 kHz), and the ^1^H MAS spectrum (at 35 kHz) is plotted along the F1 axis dimension. Extracted rows are shown [35]. (Reprinted with permission from F. G. Vogt, M. Strohmeier, Mol. Pharm. **2012**, 9, 3357. Copyright © 2012, American Chemical Society).

On a complex made by diflunisal and β-CD (Figure 10a), a 2D ^1^H-^19^F CP-HETCOR spectrum was performed to verify the incorporation of the drug in the cavities (Figure 10b). In the spectrum, the two components representing the included and free diflunisal can be clearly distinguished. A strong correlation arises between the aliphatic β-CD protons (3.5 ppm) and the more deshielded fluorine position at approximately −111 ppm. Within the included diflunisal, the expected correlation between aromatic proton positions and fluorine positions is more difficult to observe in the 2D contour plot because of a combination of the low concentration of this component and the loss of signal from spin diffusion to aliphatic β-CD protons. However, the presence of this expected correlation is evidenced by a deshielded shoulder between −105 and −110 ppm in the 1D row extraction shown for the free component [35].

^19^F nucleus was also used as a spy to get insights into the interactions of a drug (lansoprazole, LPZ, which contains a single CF_3_ group) loaded in a CD-based MOF, namely γ-CD-MOF. ^1^H-^19^F-^13^C double CP experiments provided the selection of the carbon atoms in the proximity of the fluorine atom. The resulting ^13^C NMR spectrum is compared to the ^1^H-^13^C CP NMR spectrum, in which all ^13^C atoms are present (since both the drug and the CD have protons to transfer magnetization to the carbon atoms); the spectra are normalized to the CD peak at 73 ppm. In the ^19^F-^13^C CPMAS, one can notice higher intensity, as expected, as these are the closest C atoms to the ^19^F nuclei. There is also a significant signal for the ^13^C nuclei of the CD, indicating its close spatial proximity to the drug. Among the ^13^C of the CD, the one labeled C6 (which corresponds to the CH_2_OH) has higher intensity than the other CD carbons. This indicates that the CF_3_ group is in close contact to the CH_2_OH; hence, it is located outside the CD. In combination with DFT simulation, the data indicated the formation of a 2:1 γ-CD:lansoprazole complex [36].

**Figure 11:**
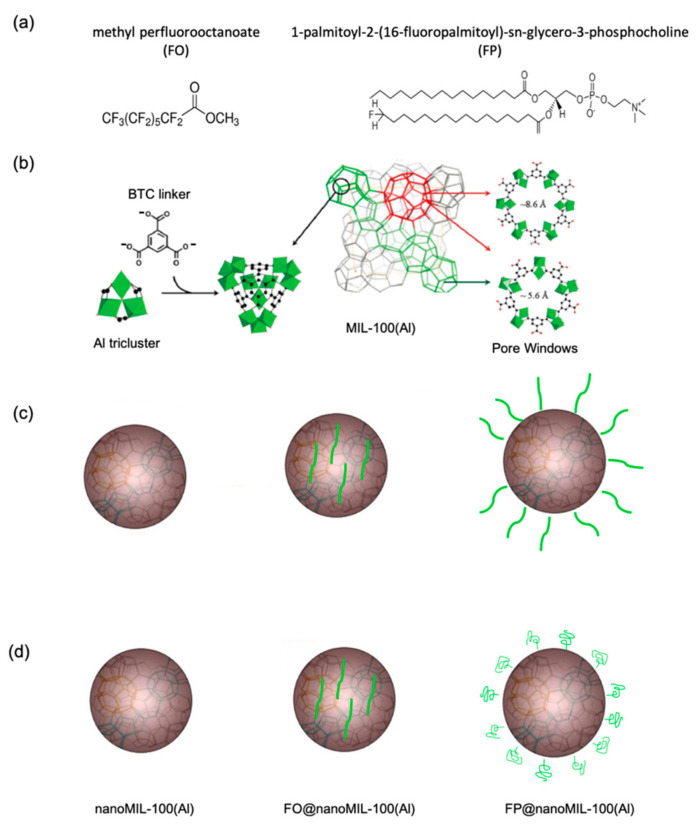
(**a**) Chemical structure of methyl perfluorooctanoate FO (middle) and 1-palmitoyl-2-(16-fluoropalmitoyl)-sn-glycero-3-phosphocholine FP (right). (**b**): Structure of MIL-100 (Al) based on the association of BTC linker and Al triclusters. The pore openings are shown on the right part. (**c**): Schematic structure of nanoMIL-100(Al) (left) and hypothesized localization of FO (middle) and FP (right) inside and outside the nanoMOFs, respectively. (**d**) Schematic structure of nanoMIL-100 (left) and localization of FO (middle) and FP (right) inside and outside the nanoMOFs, respectively, as deduced from the NMR data [37].

**Figure 12:**
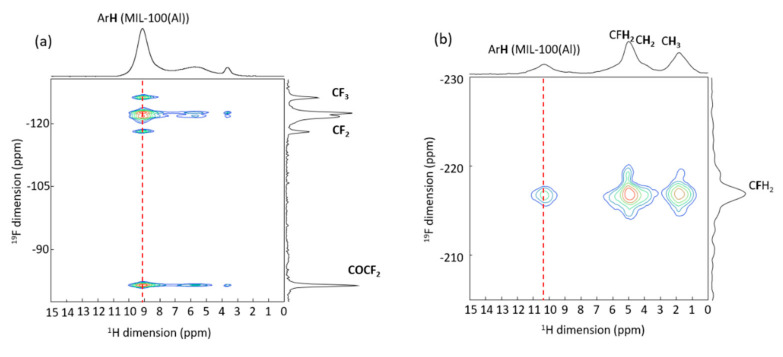
^19^F-^1^H 2D CPMAS NMR spectra of (**a**) FO@nanoMIL-100(Al) and (**b**) FP@nanoMIL-100(Al). The lines are assigned. The red dash line indicates the spatial proximity between the ^19^F resonances and the ^1^H of the MOF [37].

^19^F nucleus was also used to try to distinguish between outer and inner surface interactions. This investigation was made on MIL-100(Al) nanoparticles, which were selected because they are the diamagnetic analogues of MIL-100(Fe). The nanoMIL-100(Al) was impregnated with two different F-labeled lipid conjugates: methyl perfluorooctanoate (FO), which is supposedly small enough to enter in the pores of the MOF, and 1-palmitoyl-2-(16-fluoropalmitoyl)-sn-glycero-3-phosphocholine (FP), which is supposedly too large to enter in the pores of the MOF (Figure 11a,b). ^1^H-^19^F CPMAS 2D correlation experiments were performed on the two fluorinated-lipid nanoMIL-100(Al) materials to provide unambiguous localization of the F-lipids inside and outside the MOF. Figure 12a shows the ^19^F-^1^H 2D NMR spectrum of FO@nanoMIL-100(Al). One can see correlation peaks of strong intensity between all fluorine atoms of the lipid and the trimesate protons. This unambiguously confirms the presence of the FO lipids in the bulk of the particles, i.e., in the pores of the MOF. In the ^19^F-^1^H 2D NMR spectrum of FP@nanoMIL-100(Al) (Figure 12b), cross-peaks of strong intensity are observed between the CFH_2_ group and its neighboring CH_2_ groups from the lipid. The cross-peak between the CFH_2_ of the lipid and the proton of the trimesate has very low intensity compared to the ones of the first sample, confirming its localization on the surface of the particle, and not inside the pores. The fact that these cross-peaks are observed shows that the F-H_trimesate_ distance is not very long, which in turn could indicate that the FP is folded on the nanoparticle surface (Figure 11d) and does not stand in a brush-like manner as was initially expected (Figure 11c) [37].

For mesoporous silicas, ^29^Si proves an interesting nucleus. It is quite useful to study the influence of mesoporous structure on the uptake of the drug [38], on the interactions drug–silica [39,40], on the drug delivery properties [41] and to study the different connectivity in the silica network [42] and explore the proton chemical environments around the silica [43].

Zeolites are also potential drug carriers; a ^1^H–^29^Si HETCOR spectrum of a zeolite beta-based drug formulation containing Ag and sulfadiazine (SD) gives details into the incorporation of the drug within the zeolite matrix. The strong correlation peak detected between the Si(OAl) sites and SD aromatic and NH protons evidences the localization of the drug near the Q^4^ structures. A second correlation peak with lower intensity, is observed between the aromatic protons of SD and the signal, corresponding to [Si(1OH) + Si(1Al)] sites, at 103 ppm [44].

**Figure 13:**
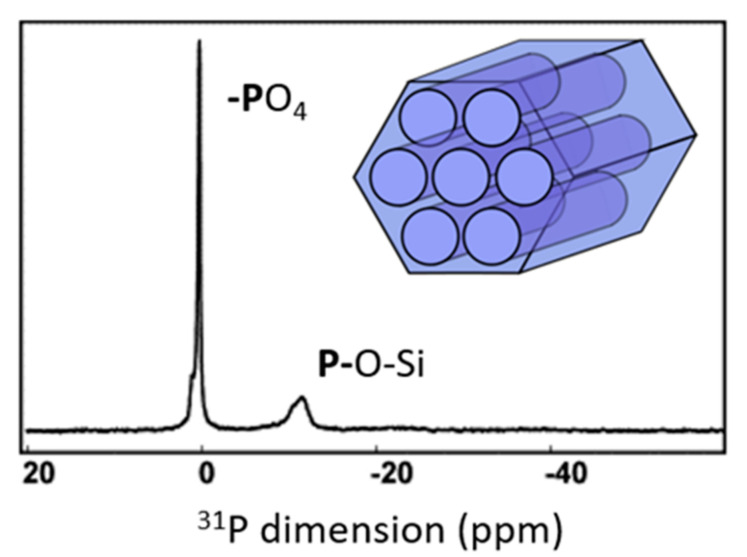
^31^P MAS-NMR spectrum of MCM-41 phosphate [46].

Numerous drugs contain a phosphorous atom in the form of phosphonate, phosphinate, or phosphate groups. ^31^P NMR spectroscopy proves very sensitive to hydrogen bonds [45]. MCM-41 containing phosphorous atoms (P-MCM-41) was used as bioactive material. The ^31^P MAS-NMR experiment (Figure 13) was performed to evaluate the amount of phosphorous and its impact in the structure of the material. In the spectrum, two groups of signals around 0 and -11 ppm are shown. Despite the low amount of phosphorus (< 1%), they could be assigned, according to phosphate units PO_4_ not bonded to silicon (called Q_0_ species) or bonded to one silicon atom (called Q_1_ species) through one P-O-Si bond. The relative intensities between both signals give a Q_0_/Q_1_m molar ratio = 1:2, which indicates that at least 66% of the P atoms are bonded to the silica framework [46].

## 6. X-Y NMR

If all the examples reported in the previous sections show the potential of the ^1^H nucleus in the study of DDS, this nucleus still sometimes leads to such complicated NMR spectra that it becomes difficult to extract information from them. In that case, although it is more demanding, it might be interesting to use pairs of heteronuclei X-Y.

### 6.1. ^27^Al-^31^P

**Figure 14:**
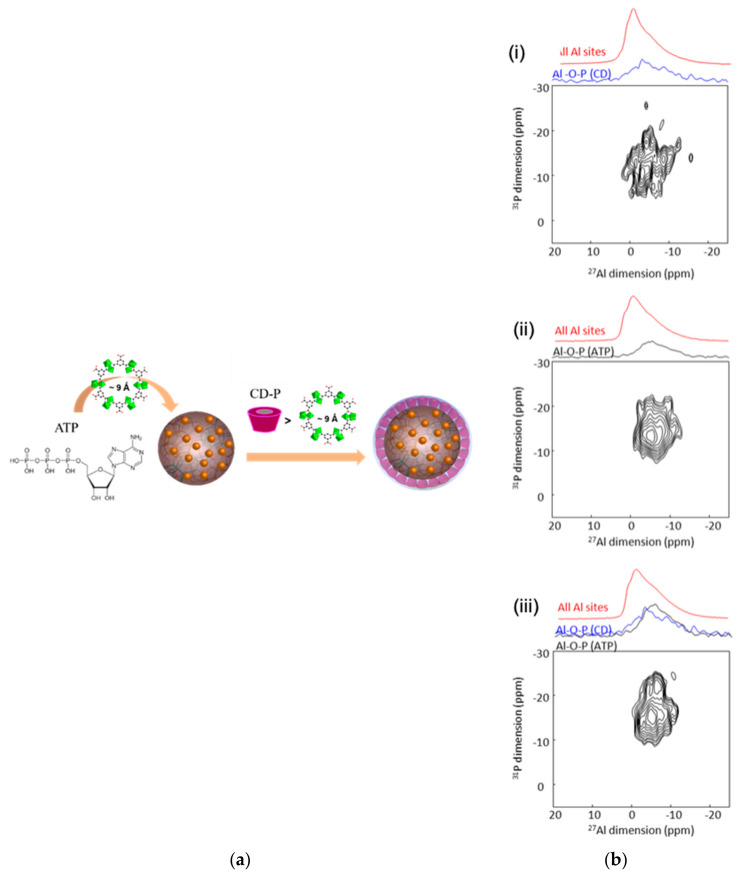
(**a**) Schematic representation of the highly porous MIL-100 (Al) nanoparticles loaded with ATP and then coated with CD-P; (**b**) ^27^Al{^31^P} MAS D-HMQC NMR spectra of CD-P coated (**i**), ATP loaded (**ii**), and CD-P coated ATP loaded nanoMIL100(Al) (**iii**). The top blue spectra are the full projections on the horizontal dimension for the surface sites, the black spectra are the full projection for the interphase sites, while the red spectra are the MAS NMR spectra shown for comparison [48].

These two nuclei happened to be present in Al-based MOFs loaded with phosphate drugs, or which had a surface covered with phosphate molecules (Figure 14a). These MOF carriers were selected because of the known affinity between aluminum and phosphorus (e.g., as in aluminophosphates), which could lead to strongly anchored drug/covering moieties. From an NMR point of view, the numerous methods have been developed for this pair of nuclei, and the required triple resonance ^1^H-^31^P-^27^Al probes are nowadays available in most labs [47].

In the nanoMIL100(Al) coated with CD-P, the surface aluminum species of the nanoparticles (NPs), i.e., those in interaction with the covering groups, could be detected by employing the through-space dipolar-based version of the solution-NMR experiments HMQC (D-HMQC) and keeping the recoupling time short enough to ensure that only the Al in very close proximity to the ^31^P were selected. The 2D ^27^Al-^31^P MAS NMR spectrum of a CD-P-coated nanoMIL-100(Al) (Figure 14b(i)) showed a ^27^Al NMR signal at −5 ppm, i.e., at a chemical shift that is close to the one observed in aluminophosphate species. This indicated the formation of an Al-O-P bond between the Al surface sites of the nanoMOF and the terminal phosphate groups of the CD-P, which very likely replace a water molecule. The same experiment was made for the ATP loaded nanoMIL-100 (Al) (Figure 14b(ii)). A six-fold coordinated ^27^Al resonance around −7 ppm was identified and suggested a close proximity (formation of an Al-O-P chemical bond) between Al species of the MOF framework and the terminal phosphate of the drug. This ^27^Al resonance is the signature of the grafted aluminum sites inside the pores of the MOF. Finally, the similarity of the spectra of ATP loaded nanoMIL-100(Al) and the target CD-P coated nanoMIL-100(Al) loaded with ATP (Figure 14b(iii)) clearly confirms that the CD-P coating on the external surface of the ATP loaded nanoMOF did not affect the Al-O-ATP bond formed inside the MOF cavities [48]. These experiments confirmed the assumption of strong affinity between aluminum and phosphorus species.

### 6.2. ^27^Al-^13^C

**Figure 15:**
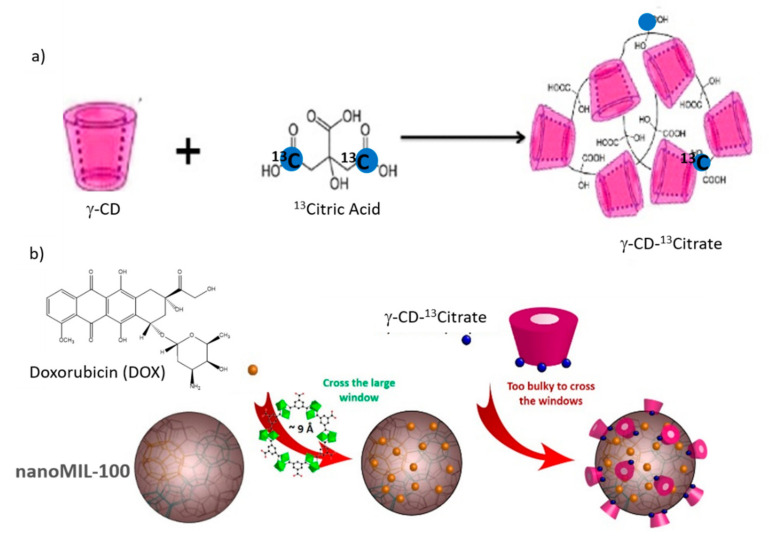
(**a**) Synthesis of γCD-^13^citrate polymers (CD-^13^CO); (**b**) Schematic representation of the highly porous MIL-100(Al) nanoparticles loaded with DOX and then coated with CD-^13^CO [48].

**Figure 16:**
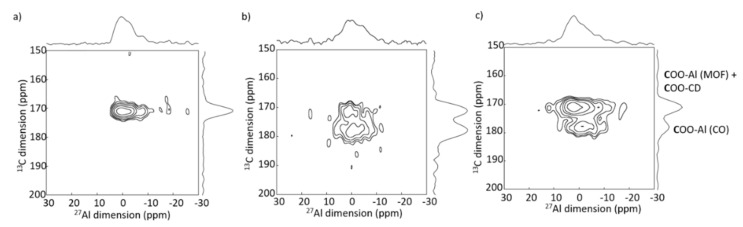
2D ^13^C-^27^Al D-HMQC 2D of (**b**) CD-^13^CO@nanoMIL-100(Al) and (**c**) DOX loaded CD-^13^CO@nanoMIL-100(Al). The spectrum recorded on pure (**a**) nanoMIL-100(Al) is shown for comparison [49].

^13^C-^27^Al 2D MAS NMR experiments were performed to study drug carrier interactions and obtain information about the localization of the drug [49]. Oligomers based on CD cross-linked with citric acid (CD-CO) were shown to interact strongly with the anticancer drug, Doxorubicin (DOX) [50,51]. DOX was also shown to enter in the pores of nanoMIL-100 (Al) [46]. Therefore, this CD-CO water soluble oligomer was considered as a versatile coating to promote DOX incorporation and control its release. To study the interactions taking place in this system and have selectivity between the bulk and the surface, the coating was synthesized using a ^13^C label citric acid (1,5-^13^C_2_ citric acid). The obtained CD-^13^CO oligomer was used to cover the surface of the nanoMIL-100(Al) after loading of DOX drug in the pores (Figure 15) [49]. To understand the interaction between the CD-CO coating and the MOF nanoparticles, 2D ^13^C-^27^Al MAS correlation NMR experiments were performed (Figure 16), showing the spatial proximity between carbon and aluminum atoms. On the 2D NMR spectrum of CD-^13^CO@nanoMIL-100(Al) (Figure 16b), correlation peaks of strong intensity were observed between the COO of the citric acid (^13^C resonance at 180 ppm) and the surface Al sites, confirming that the CD-CO oligomer has high affinity with the NP surface. Note that the ^13^C resonance at 175 ppm contains both the carboxylic group of the trimesate linker of the MOF (not labeled but present in large quantity) and the COO-CD of the CD-^13^CO coating. The same experiment was performed on the DOX loaded CD-^13^CO@nanoMIl-100(Al) (Figure 16c) and shows that the coating is still in strong interaction with the NP surface. One can notice a change of the relative intensity between the two carbon resonances. This indicates that in addition to going in the pores of the MOF, the DOX molecules also interact significantly with the CD-CO coating. Notably, since the intensity of ^13^C resonance at 180 ppm has decreased, it very likely indicates that part of the ^13^COO-Al bonds formed between the citric acid moieties and the surface Al sites have been broken, probably in favor of the interaction of the citric acid with the DOX drug.

## 7. Conclusions

In this review, we have illustrated different approaches used during the years to analyze the supramolecular structures of DDS by ssNMR spectroscopy. This technique proves very useful to study the confinement of the drug in the nanoparticulate system, its interaction with the host matrix, and its release. Moreover, ssNMR spectroscopy gave invaluable information on the location of the shell, possible penetration inside the nanoparticles’ pores, and its interaction with the core. The easiest and most analyzed nucleus is ^1^H, but sometimes, the systems are so complex that the ^1^H 1D NMR spectra result in a broad signal without any information available. Several strategies can be adopted in that case. The first one is to perform ^1^H-^1^H 2D NMR experiments, increasing the resolution and having more information thanks to the non-direct dimension. As an alternative, heteronuclei (X) NMR, such as ^13^C, ^29^Si, ^27^Al, ^31^P, and ^19^F present on the drug or the delivery system can be used in conjunction with proton NMR in ^1^H-X NMR experiments. Finally, when proton NMR is not informative at all, NMR of pairs of heteronuclei X-Y can be used. With this complete set of NMR tools and methods, the supramolecular structures of a large variety of DDSs, as illustrated in this review, have been studied. This deep characterization step is essential to guide the design of more performant DDSs. Although not shown here, ssNMR, in complement with solution state NMR, can also be used to study both the degradation of the particles and drug release.
